# Adolescents' experience of comments about their weight – prevalence, accuracy and effects on weight misperception

**DOI:** 10.1186/1471-2458-9-271

**Published:** 2009-07-30

**Authors:** Wing-Sze Lo, Sai-Yin Ho, Kwok-Kei Mak, Yuen-Kwan Lai, Tai-Hing Lam

**Affiliations:** 1 Department of Community Medicine and School of Public Health, The University of Hong Kong, Hong Kong SAR, PR China

## Abstract

**Background:**

Weight comments are commonly received by adolescents, but the accuracy of the comments and their effects on weight misperception are unclear. We assessed the prevalence and accuracy of weight comments received by Chinese adolescents from different sources and their relation to weight misperception.

**Methods:**

In the Hong Kong Student Obesity Surveillance (HKSOS) project 2006–07, 22612 students aged 11–18 (41.5% boys) completed a questionnaire on obesity. Students responded if family members, peers and professionals had seriously commented over the past 30 days that they were "too fat" or "too thin" in two separate questions. The accuracy of the comments was judged against the actual weight status derived from self-reported height and weight. Self-perceived weight status was also reported and any discordance with the actual weight status denoted weight misperception. Logistic regression yielded adjusted odd ratios for weight misperception by the type of weight comments received.

**Results:**

One in three students received weight comments, and the mother was the most common source of weight comments. Health professional was the most accurate source of weight comments, yet less than half the comments were correct. Adolescents receiving incorrect comments had increased risk of having weight misperception in all weight status groups. Receiving conflicting comments was positively associated with weight misperception among normal weight adolescents. In contrast, underweight and overweight/obese adolescents receiving correct weight comments were less likely to have weight misperception.

**Conclusion:**

Weight comments, mostly incorrect, were commonly received by Chinese adolescents in Hong Kong, and such incorrect comments were associated with weight misperception.

## Background

It is well known that many adolescents misperceive their weight [1-8]. For example, Brener *et al*. [2] reported that half the normal weight high school students had weight misperception. About 30% of normal weight Hong Kong adolescents misperceive themselves as fat [9], and more girls than boys overestimate their weight [4,10-14]. The perception of suboptimal weight is associated with depressive symptoms and other psychological problems among adolescents in both cross-sectional and longitudinal studies [15-18].

The lack of knowledge about and access to growth charts has probably made it difficult for adolescents to evaluate their weight status objectively. Frequent exposure to the media's portrayals of thin ideal for females [19-23] and muscular physique for males [24-26] may therefore predispose adolescents to weight misperception [27-29]. The Tripartite Influence Model [30,31] also suggests that weight comments and opinions from parents and peers may influence adolescent weight perception.

Teasing about weight is common among adolescents [32-34]. Neumark-Sztainer *et al*. [32] reported in a large US study that 25% of secondary school students were teased about their weight several times in the past year. Adolescents are sensitive to weight-related influences, and may experience tremendous pressure from weight teasing. Cross-sectional and retrospective studies [30,32-36] have linked adolescent weight teasing to poor self-esteem and body image, unhealthy weight-control attempts, as well as eating disorders. There is also prospective evidence that weight teasing predicts psychological distress among adolescents [37].

Existing Western studies were implicit whether weight teasing was about being too fat or too thin, but given the high prevalence of obesity and the associated negative image, the former was much more likely. Although the effects of teasing about being too thin are uncertain, the perception of being too thin is associated with anxiety and depressive symptoms in cross-sectional studies [16,18]. Weight teasing about thinness would be more relevant in developing countries such as China, where underweight is common [38].

Most studies about adolescent weight teasing referred to that from peers and parents [32-35]. However, in Asian 3-generation families, grandparents may also exert great influence on adolescent eating patterns [39]. Grandparents, teachers, social workers and health professionals are all potential sources of adolescent weight comments although little is known about the prevalence, accuracy and effects of these comments.

Previous studies mainly focused on weight teasing, which included disparaging nicknames and making fun of others' weight and body shape [32-34,40]. However, weight-related comments could also be constructive and well-intentioned. For example, a caring mother could remind her teenage son of his bulging waistline and a family doctor could and should advise adolescents of their weight status. Moreover, to the best of our knowledge, no study has examined whether correct, incorrect or even conflicting (same person receiving opposite weight comments of being too fat and too thin) weight comments are independently associated with weight misperception in adolescents.

In the present study we extended existing research in three ways. First, we included parents, siblings and grandparents, as well as teachers, social workers and health professionals as separate sources of weight comments. Second, we examined the accuracy of those weight comments. Third, we investigated the effects of different types (correct, incorrect and conflicting) of weight comments on weight misperception. We hypothesized that adolescents who received incorrect and conflicting weight comments were more likely to have weight misperception, whereas those who received correct weight comments were less likely to have weight misperception, compared with adolescents who did not receive any weight comments over the past 30 days.

## Methods

### Data collection and subjects

The present study was part of a large population-based study, the Hong Kong Student Obesity Surveillance (HKSOS) project. Stratified cluster sampling was applied, and the schools were sampled with stratification by school district, source of funding, language of instruction (Chinese/English), religious background (Christian/Others/None) and single sex/co-education to represent all main stream non-international secondary schools in Hong Kong. Forty-two schools participated in this survey. All Form 1 to 7 students (equivalent to Grade 7–12 in US) in selected schools were invited to participate. We have obtained the consent of schools who acted *in loco parentis *for the students. Passive consent from the parents was obtained and all students participated on voluntary basis. Ethical approval was granted by the Institutional Review Board of the University of Hong Kong/Hospital Authority Hong Kong West Cluster.

In 2006–2007, the anonymous baseline survey was self-administered in classrooms under the supervision of trained researchers or teachers. In the present study 31603 students aged 11 to 18 were eligible. Of these, 2319 were excluded because self-reported height and weight data were missing. As the maximum reference values for height of the Hong Kong official weight-for-height (WFH) cutoffs were 175 cm in boys and 165 cm in girls [41], students who exceeded these height limits were excluded (n = 738). Also excluded were extreme body mass index (BMI) values beyond 10 (biological limit) and 50 (morbidly obese) (n = 4752) [42-44]. After further exclusion of 1182 questionnaires with incomplete data, 22612 (41.5% boys) remained for analyses. The test-retest reliability of measures used in this study was assessed with 1147 students (31.3% boys; mean age = 14.8 ± 1.6) from 3 of the participating schools over an interval of one month.

### Measures

#### Actual weight status

Height (cm/inch) and weight (kg/lb) were self-reported by the participants to the nearest integer. Using sex-specific Hong Kong official weight-for-height (WFH) cutoffs [41], participants were defined as underweight (< 80% median weight-for-height), normal weight (80–120% median weight-for-height) and overweight/obese (> 120% median weight-for-height).

#### Weight perception

Weight perception was measured using a standard question that asked students to describe their weight status as very thin, thin, just right, fat or very fat. This measure has been widely used in population surveys conducted in the US [26] and the UK [27], as well as in a large adolescent health behaviour study in Mainland China [4]. As relatively few students chose extreme categories (6.4% for very fat and 4.5% for very thin), the five categories were consolidated into three groups: thin (including very thin), just right and fat (including very fat). Overweight/obese, underweight and normal weight students who considered themselves other than fat, thin or just right, respectively, were classified as having "weight misperception" as opposed to "correct perception".

#### Prevalence of weight comments received

To assess the prevalence of weight comments received by the students, two questions were used. The first question asked whether anyone had seriously commented over the past 30 days that the student was "too fat". A list of social contacts was provided as response options including: 1) family members (father, mother, siblings, grandfather, grandmother and other relatives); 2) peers (friends and classmates); 3) professionals (teachers, social workers and health professionals); and 4) others (domestic helpers and neighbors). There was also an option of "none" to indicate that no one had given such comments. The second question was identical except that "too fat" was replaced by "too thin".

#### Accuracy and types of weight comments

The accuracy of the weight comments was assessed against the actual weight status. The weight comment of "too fat" was deemed correct to overweight/obese students but incorrect to normal or underweight students. Similarly, "too thin" was considered a correct comment to underweight students but incorrect to overweight/obese or normal weight students. Weight comments of "too fat" or "too thin" were considered incorrect to normal weight students. Therefore, according to the weight comments received and their accuracy, four categories were identified: 1) no weight comments, 2) correct weight comments, 3) incorrect weight comments and 4) conflicting weight comments (receiving both "too fat" and "too thin" comments regardless of actual weight status).

### Data analysis

#### Bivariate and multivariate analyses

Chi-square and Student's t statistics were used to test the differences of basic characteristics and weight comments between boys and girls. The prevalence of weight comments from different sources was also compared between sexes by Chi-square statistics. In the bivariate analysis, the percentages of adolescents receiving correct, incorrect, conflicting or no weight comments were calculated, and Chi-square statistics were used to compare between correct perception and weight misperception.

In the multivariate logistic regression analyses, the binary outcome variable of weight misperception was regressed on the independent variable of weight comments taking the category "none" (no weight comments received) as the reference group. Odds ratios (ORs) and 95% confidence intervals (95% CI) for weight misperception were estimated, adjusting for age (as a continuous variables), BMI (weight (kg) divided by height in squared (m^2^)), and three socio-demographic factors, including the place of birth (Hong Kong, or elsewhere), the highest parental education (≤ primary, secondary, or ≥ tertiary), and perceived family affluence (relatively poor, medium, or relatively wealthy). The regression model was conducted with robust standard errors accounting for school clustering effect (design effect = 3.16). A CI range excluding 1 and p < 0.05 indicated that the OR was significant. Correlation coefficients between independent variables were examined (r = 0.007 to 0.44), and none of which exceeded 0.8 that indicates problems of multicollinearity [45]. All the main analyses were stratified by sex and actual weight status.

All statistical analyses were performed using STATA 9.0 (Stata Corporation, College Station, TX) with the significance level set at 5%. Multilevel logistic regression was not performed as we aimed to investigate population-averaged effects rather than school-specific effects.

#### Test-retest reliability

Intra-class correlation coefficients (ICC) were used to examine the test-retest reliability of continuous variables [46], while Kappa statistics (κ) was used for categorical variables. When there is a low prevalence of a particular response, or when the frequency is unbalanced, κ may be low [47], therefore percent agreement was also examined for categorical variables. The ICC for height (0.95) and weight (0.85) data in 1147 subjects were high and comparable to those of published reports [48]. Others have also found high correlations between self-reported and measured anthropometric data in adolescents [49]. Based on percent agreement, test-retest reliability of weight comments ranged from moderate to high (κ = 0.16 – 0.60; percent agreement = 74.3% – 95.1%). Agreements of perceived weight status (κ = 0.68; percent agreement = 79.9%) was good. These test-retest reliability statistics were similar between boys and girls.

#### Handling of missing data

Missing values for the place of birth (0.6%), highest parental education (12.8%) and housing type (1.0%) were imputed using multiple imputations [50]. Five imputations were generated using the software program Amelia, based on a model that uses values from other variables to achieve optimal estimates [38]. Similar results were obtained using the imputed or original databases, so only the imputed database was used for its larger effective sample size.

## Results

### Basic characteristics of our subjects

Table 1 summarizes the basic characteristics of the final sample (n = 22612; 41.5% boys). The sample was representative of Hong Kong adolescents in terms of sex, age, and residential district (all Cohen effect sizes [51] ≤ 0.2) (table not shown) despite some subjects were excluded due to missing data.

**Table 1:** Basic characteristics of participants (N = 22612)

Characteristics	All (N = 22612)	Boys (N = 9375)	Girls (N = 13237)	Test statistics
Age (years, mean, SD)	14.7 (1.70)	14.6 (1.70)	14.7 (1.70)	t = -3.35, p = 0.019
Form (%)				χ^2 ^= 27.52, p < 0.001
Junior (F1–F3)^a^	59.6	61.7	58.2	
Senior (F4–F7)^a^	40.4	38.3	41.8	
BMI (kg/m^2^, mean, SD)	19.2 (2.99)	19.5 (3.29)	18.9 (2.73)	t = 13.29, p < 0.001
Weight status by local references (%)				χ^2 ^= 204.15, p < 0.001
Underweight	8.7	8.6	8.7	
Normal weight	79.0	75.3	81.6	
Overweight/Obese	12.4	16.1	9.7	
Parental education level (%)				χ^2 ^= 26.07, p < 0.001
Primary or below	12.0	12.2	11.9	
Secondary	65.9	64.1	67.2	
Tertiary or above	22.1	23.7	21.0	
Place of birth (%)				χ^2 ^= 3.63, p = 0.06
Hong Kong	73.9	73.6	74.6	
Other places^b^	26.1	26.4	25.4	
Family affluence (%)				χ^2 ^= 38.03, p < 0.001
Relatively poor	36.4	38.6	34.9	
Medium	52.6	50.3	54.3	
Relatively wealthy	10.9	11.1	10.8	
Weight perception (%)				χ^2 ^= 177.35, p < 0.001
Correct perception	47.6	52.9	43.9	
Weight misperception	52.4	47.1	56.1	
Received weight comments of being "too fat"	29.0	22.3	33.8	χ^2 ^= 350.00, p < 0.001
Received weight comments of being "too thin"	26.9	28.4	25.8	χ^2 ^= 17.71, p < 0.001

^a ^F1–F3 is equivalent to grade 7 to grade 9; F4–F7 is equivalent to grade 10 to grade 12.

^b ^Other places: Mainland China (majority), Macau, Western countries and others.

The prevalence rates of underweight and overweight/obese were 8.7% (8.6% for boys, 8.7% for girls) and 12.4% (16.1% for boys, 9.7% for girls), respectively. Boys and girls were significantly different in terms of age, form (school grade), BMI, weight status, highest parental education, place of birth, and perceived family affluence.

Weight misperception was identified in over half the subjects (52.4%) and was more common in girls (56.1%) than boys (47.1%) (χ^2 ^= 177.4, p < 0.001). In the past 30 days, 29.0% and 26.9% of adolescents received weight comments of being "too fat" and "too thin", respectively. The comment of being "too fat" was more commonly received by girls (33.8%) than boys (22.3%) (χ^2 ^= 350.0, p < 0.001), while that of "too thin" more commonly received by boys (28.4%) than girls (25.8%) (χ^2 ^= 17.7, p < 0.001).

### Sources of "too fat" and "too thin" weight comments by sex

Among adolescents who received comments of being "too fat", 53.9% of the comments came from a single source, 24.7% from two sources and 10.6% from three sources. The corresponding figures for comments of being "too thin" were 54.2%, 22.7% and 10.2%.

Table 2 shows that the mother was the most common source of "too fat" comments for both boys and girls, followed by siblings and classmates. The comment of being "too fat" was more commonly received by girls than boys from all sources except the grandfather although more boys than girls were overweight/obese. The mother was also the most common source of "too thin" comments. Girls were nearly twice as likely as boys to receive comments of being "too thin" from classmates and friends. Grandparents, teachers, social workers and health professionals were more likely to give comments of being "too thin" than "too fat" to both boys and girls.

**Table 2:** Sources of weight comments received by boys and girls

	**Too fat (%)**				**Too thin (%)**				**Ratio** (Too fat/Too thin)	
	
	Boys	Girls	χ^2^	p	Boys	Girls	χ^2^	P	Boys	Girls
Family	14.4	26.1	446.21	< 0.001	22.2	18.9	37.35	< 0.001	0.65	1.38
Father	5.3	7.7	50.98	< 0.001	8.7	7.1	20.86	< 0.001	0.61	1.08
Mother	9.0	17.0	299.75	< 0.001	15.1	12.4	36.43	< 0.001	0.60	1.37
Siblings	4.1	10.6	312.18	< 0.001	3.9	2.8	20.41	< 0.001	1.05	3.79
Grandfather	1.0	0.7	5.09	0.024	2.0	1.3	18.53	< 0.001	0.50	0.54
Grandmother	0.6	1.5	41.33	< 0.001	2.5	2.7	0.56	0.45	0.24	0.56
Other relatives	1.2	4.4	185.95	< 0.001	3.6	4.4	6.88	0.009	0.33	1.00
Peers	7.5	13.5	204.17	< 0.001	6.5	11.4	153.34	< 0.001	1.15	1.18
Classmates	5.2	8.4	89.30	< 0.001	4.7	7.8	84.15	< 0.001	1.11	1.08
Friends	4.2	8.9	189.96	< 0.001	4.2	7.7	112.95	< 0.001	1.00	1.16
Professionals	2.1	2.2	0.62	0.50	2.8	2.8	0.007	0.97	0.75	0.79
Teachers	0.8	1.0	3.37	0.07	1.3	1.4	0.24	0.63	0.62	0.71
Social workers	0.4	0.4	0.09	0.77	0.5	0.6	0.75	0.39	0.80	0.67
Health professionals	0.9	0.9	0.19	0.89	1.2	1.2	0.03	0.87	0.75	0.75
Others^a^	5.5	4.7	5.17	0.012	6.5	4.5	42.99	< 0.001	0.85	1.04

^a ^Others include neighbors and domestic helpers.

### Prevalence of correct weight comments from different sources

**Figure 1:**
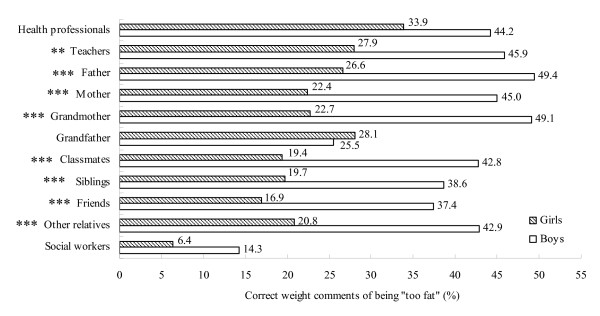
**Prevalence of correct weight comments of being "too fat" by source and sex (descending order)**. Key: *p < 0.05; **p < 0.01; ***p < 0.001 between boys and girls.

**Figure 2:**
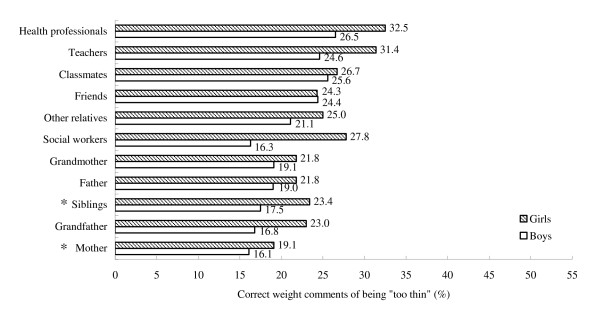
**Prevalence of correct weight comments of being "too thin" by source and sex (descending order)**. Key: *p < 0.05; **p < 0.01; ***p < 0.001 between boys and girls.

Figures 1 and 2 show the prevalence of correct weight comments from different sources. In general, health professionals and teachers were the two most accurate sources of "too fat" and "too thin" comments. The third most accurate source of "too fat" comments was the father, and of "too thin" comments classmates.

Boys received more correct comments of being "too fat" than girls from all sources but the grandfather (28.1% girls vs 25.5% boys, χ^2 ^= 0.2, p = 0.69). The accuracy of "too thin" comments from all sources was similar in boys and girls except that it was more accurate for girls than boys of comments from the mother (19.1% girls vs 16.1% boys, χ^2 ^= 4.8, p = 0.029) and siblings (23.4% girls vs 17.5% boys, χ^2 ^= 3.9, p = 0.049).

### Bivariate analyses

Before examining the associations between the accuracy of weight comments and weight misperception, the weight comments from different sources were combined as the sample size of each source was too small. Among those who received weight comments in the past 30 days (47.8% in total), 62.8% were incorrect comments, 17.1% were conflicting comments and only 20.1% were correct comments.

Table 3 shows the bivariate relations between the accuracy of weight comments received and weight perception, stratified by sex and actual weight status. In both boys and girls, the accuracy of weight comments received was significantly different between those with correct perception and weight misperception, among all weight status groups (all p < 0.001). As the accuracy of weight comments was significantly associated with weight perception, logistic regression models were then performed to estimate the OR and (95% CI) of having weight misperception, adjusting for potential confounders.

**Table 3:** The types of weight comments by weight perception in boys and girls of different weight status^a^

	Boys (n = 9375)				Girls (n = 13237)			
	
	Correct perception (n = 4958) %	Weight misperception (n = 4417) %	χ^2^	p	Correct perception (n = 5812) %	Weight misperception (n = 7425) %	χ^2^	p
UN	(n = 496)	(n = 311)	135.45	< 0.001	(n = 612)	(n = 539)	246.93	< 0.001
No comments	38.5	67.5			26.6	51.6		
Correct	54.6	15.1			65.7	22.3		
Incorrect	0	3.5			1.0	15.0		
Conflicting	6.9	13.8			6.7	11.1		
NW	(n = 3353)	(n = 3710)	545.23	< 0.001	(n = 4082)	(n = 6715)	600.24	< 0.001
No comments	75.7	50.3			65.5	41.6		
Correct	-	-			-	-		
Incorrect	16.2	40.6			27.8	50.3		
Conflicting	8.1	9.1			6.7	8.0		
OV/OB	(n = 1109)	(n = 396)	261.11	< 0.001	(n = 1118)	(n = 171)	228.84	< 0.001
No comments	37.9	62.1			30.5	53.8		
Correct	51.2	8.8			62.8	17.0		
Incorrect	2.0	13.9			1.0	19.3		
Conflicting	8.9	15.2			5.7	9.9		

^a ^Using the sex-specific local weight-for-height (WFH) cutoffs, our subjects were defined as underweight (< 80% median WFH), normal weight (80% – 120% median WFH) and overweight/obese (> 120% median WFH).

UN = underweight; NW = normal weight; OV/OB = overweight/obese

### Multivariate analyses

Table 4 shows the adjusted ORs for weight misperception by the accuracy of weight comments received compared with not receiving any weight comments. ORs greater than 1 indicate that weight misperception is more likely. Incorrect weight comments were positively associated with weight misperception in normal weight and overweight/obese boys. ORs could not be calculated for underweight boys as none of them who had correct weight perception received incorrect weight comments. Conflicting comments were associated with an increased risk of weight misperception (OR = 1.73, 95% CI = 1.43–2.09, p < 0.001) among boys who were normal weight but not underweight or overweight/obese. Underweight and overweight/obese boys who received correct weight comments were 84% and 89%, respectively, less likely to have weight misperception, thus indicating a beneficial effect.

**Table 4:** Adjusted odds ratio (95% confidence interval) for weight misperception in relation to different types of weight comments received by sex and weight status^a ^(N = 22612)

		Boys		Girls	
	**Types of weight comments received**	Adjusted^b^	p	Adjusted^b^	p

**UW**	None	1		1	
	Correct	0.16 (0.11–0.22)	< 0.001	0.18 (0.14–0.23)	< 0.001
	Incorrect^c^	-	-	6.88 (2.68–17.68)	< 0.001
	Conflicting	1.25 (0.80–1.94)	0.3	0.74 (0.45–1.20)	0.2
					
**NW**	None	1		1	
	Correct	-	-	-	-
	Incorrect	3.73 (3.28–4.23)	< 0.001	2.89 (2.67–3.13)	< 0.001
	Conflicting	1.73 (1.43–2.09)	< 0.001	1.93 (1.67–2.23)	< 0.001
					
**OV/OB**	None	1		1	
	Correct	0.11 (0.08–0.15)	< 0.001	0.17 (0.11–0.26)	< 0.001
	Incorrect	4.75 (2.79–8.09)	< 0.001	12.7 (5.24–30.83)	< 0.001
	Conflicting	1.07 (0.80–1.44)	0.7	0.97 (0.53–1.79)	0.9

^a ^Using the sex-specific local weight-for-height (WFH) cutoffs, our subjects were defined as underweight (< 80% median WFH), normal weight (80% – 120% median WFH) and overweight/obese (> 120% median WFH).

UW = Underweight; NW = Normal weight; OV/OB = Overweight/Obese.

^b ^Adjusted for age, highest parental education, family affluence, place of birth, BMI and school effect.

^c ^OR cannot be calculated due to the insufficient number in the group (Underweight boys with weight misperception did not receive any incorrect comments).

Similar results were observed in girls. Girls who received incorrect weight comments were more likely to have weight misperception regardless of their weight status. The small numbers of underweight and overweight/obese girls have resulted in the wide 95% CI in these subgroups (only 1% of girls with correct weight perception received incorrect weight comments, as shown in Table 3). Normal weight girls who received conflicting weight comments were also more likely to have weight misperception, with adjusted OR (95% CI) of 1.93 (1.67–2.23). However, conflicting comments in underweight and overweight/obese girls were not significantly associated with weight misperception. Consistent with the results in boys, underweight and overweight/obese girls who received correct weight comments had significantly lower risks of weight misperception, with adjusted odds ratio (95% CI) of 0.18 (0.14–0.23) and 0.17 (0.11–0.26), respectively.

## Discussion

The most important finding of the present study is that despite the high rates of weight comments received by adolescent boys and girls, less than one-fifth of the comments were accurate. This is alarming as we also found incorrect weight comments were associated with weight misperception. Underweight and overweight/obese adolescents with weight misperception may be unaware of their weight problems [8,52], whereas normal weight adolescents with distorted perceptions of their weight may engage in unhealthy weight control behaviors [52]. Previous studies have found that normal weight adolescents with weight misperception were more likely to have psychosocial health problems and poor self-esteem [4,6,53].

### Prevalence of weight comments from different sources

Our findings are in line with previous research that girls received significantly more weight comments of being fat from family members and peers than boys [32,34]. While previous research focused mainly on weight comments from the parents or the family as a group, we have specifically included the father, mother, grandfather, grandmother and siblings. We found that the comment of being "too fat" was most commonly made by the mother and siblings. It was suggested that parental weight comments would increase teasing by siblings [33] although this could not be examined using our data. We also found that girls were more likely than boys to receive comments of being "too fat" from siblings (Table 2). Although weight comments from grandparents were uncommon, they were interestingly dominated by the comment of being "too thin" (Table 2). As being fat is traditionally a sign of health and wealth among older Chinese people, many of whom have experienced poverty and hunger, grandparents may prefer children to eat and weigh more [39].

Our result that family members exceeded peers as a source of weight comments was in contrast to published findings [34]. This is probably because any well-intentioned comments, more likely from family members, were included in the present study, whereas only weight teasing was included in other studies.

### Low accuracy of weight comments by health professionals

As expected, health professionals were the most accurate source of weight comments, although less than half the comments were correct. In a US study, 76% of obese adolescents were correctly identified as obese by physicians [54]. A recent Canadian study also reported that about 60% of physicians could accurately estimate adolescent body size [55]. These findings may not be directly comparable due to differences in research methods and the proportion of borderline overweight and obese subjects whose weight status would be more difficult to determine [56]. Nevertheless, health professionals in Hong Kong should routinely measure the weight status of adolescents and advise accordingly despite the lack of consultation time, space and appropriate equipment [56]. The rapidly changing body dimensions during adolescence, and the lack of a well recognized weight status standard for children might have also contributed to the low accuracy of weight comments by health professionals.

### Prevalence of weight misperception among Hong Kong adolescents

More than half our students had weight misperception (Table 3), which is consistent with previous studies in Western countries [2,3], Mainland China [4] and Hong Kong [9]. The lack of knowledge about and access to growth charts probably make it difficult for adolescents to evaluate their weight status objectively. As adolescents are susceptible to social influences [57-59], weight comments and opinions from the family and peers may also influence weight perception according to the Tripartite Influence Model [30,31]. However, our study found that most weight comments from parents and peers were inaccurate. In Hong Kong, one in three adults misperceived their own weight status [60] while in the UK and US, only 25–35% of parents could correctly identify the weight status of their obese adolescent child [49,61]. Whether parents who misperceived their own weight status are more likely to give incorrect weight comments to their children is still unclear.

### Weight misperception and the accuracy of weight comments

We found that incorrect and conflicting weight comments were in general associated with weight misperception in adolescents. In contrast, correct weight comments were associated with correct weight perception. While weight teasing was linked to negative outcomes such as poorer self-esteem, unhealthy weight-control behaviors, depressive symptoms and even eating disorders [30,32-36], we found that accurate weight comments may help adolescents establish an appropriate weight perception. Further studies should examine whether correct weight perceptions are associated with effective weight control among adolescents.

### Strengths and limitations

Our large territory-wide sample allowed us to perform detailed analyses stratified by sex and weight status. We have also considered more sources of weight comments than previous studies and the comments were not limited to teasing only.

However, our study has several limitations. The anthropometric data and weight comments were self-reported although their test-retest reliability was good and comparable to that of other similar studies [2,49]. Weight comments were self-reported, but their association with weight misperception as expected supported the validity of the self-reported comments. To reduce any recall error of reported weight comments, a shorter time frame of the past 30 days was used. The frequency of weight comments was not assessed due to the length of the weight comments.

Our data were not sufficient to compare the impact of individual source of weight comments on weight misperception. According to Keery et al. [33], weight teasing from the father and elder brothers was associated with higher psychosocial distress than the mother and sisters. The effect of weight teasing or comments may also differ in male and female recipients. Therefore, more information about the relationship between the source and recipient of the weight comments is needed to understand the different effects. Future study should also investigate the feelings of adolescents after receiving weight comments. The association of incorrect weight comments and weight misperception with negative psychosocial health problems should be clarified by longitudinal studies.

## Conclusion

Weight comments from family members and peers are commonly received by Chinese adolescents, yet most comments are inaccurate. Family members, peers and professionals should realize the potential adverse effects of incorrect weight comments, and adolescents should be taught how to correctly assess their weight status to establish correct weight perceptions. Health professionals should regularly give appropriate weight advice to adolescents based on objective measurements.

## Competing interests

We hereby declare that we do not have a financial association or other conflict of interest with the subjects mentioned in this manuscript.

## Authors' contributions

WSL contributed to study design and management, performed statistical analyses and drafted the manuscript; SYH is the principle investigator of the HKSOS project and critically revised the manuscript; KKM and YKL contributed to study design, coordination and revision of the manuscript; THL gave critical revision of the manuscript and supervision. All authors read and approved the final manuscript.

## Pre-publication history

The pre-publication history for this paper can be accessed here:


